# Exposure to organochlorine pesticides and non-Hodgkin lymphoma: a meta-analysis of observational studies

**DOI:** 10.1038/srep25768

**Published:** 2016-05-17

**Authors:** Dan Luo, Tingting Zhou, Yun Tao, Yaqian Feng, Xiaoli Shen, Surong Mei

**Affiliations:** 1 State Key Laboratory of Environment Health (Incubation), Key Laboratory of Environment and Health, Ministry of Education, Key Laboratory of Environment and Health (Wuhan), Ministry of Environmental Protection, School of Public Health, Tongji Medical College, Huazhong University of Science and Technology, #13 Hangkong Road, Wuhan, Hubei, 430030, China

## Abstract

Growing evidence indicates that exposure to organochlorine pesticides (OCPs) could increase non-Hodgkin lymphoma (NHL) risk. However, results from epidemiological studies investigating this association remain controversial. We thus conducted a meta-analysis to quantitatively evaluate the association between OCP exposure and NHL risk. Relevant publications were searched in PubMed, Web of Science, and Embase and identified according to the inclusion criteria. Thirteen studies (6 nested case-control, 1 case-cohort, and 6 case-control) were selected for this meta-analysis. We used odds ratios (ORs) with 95% confidence intervals (CIs) to estimate the relationship between OCPs exposure and NHL risk. The summary OR for included studies was 1.40 (95% CI 1.27 to 1.56). No overall significant heterogeneity in the OR was observed (P_h_ = 0.253, I^2^ = 12.6%). Furthermore, OR estimates in subgroup analyses were discussed, and strong associations were observed for dichlorodiphenyldichloroethylene (DDE, OR = 1.38, 95% CI 1.14 to 1.66), hexachlorocyclohexane (HCH, OR = 1.42, 95% CI 1.08 to 1.87), chlordane (OR = 1.93, 95% CI 1.51 to 2.48), and hexachlorobenzene (HCB, OR = 1.54, 95% CI 1.20 to 1.99). This meta-analysis had suggested that total OCPs of interest was significantly positively associated with NHL risk.

Non-Hodgkin lymphoma (NHL), a heterogeneous group of malignancies arising from the lymphatic system, including lymph nodes and lymphoid tissues and eventually migrating to the bone marrow and blood, is the sixth most commonly diagnosed cancer in both males and females in the United States^1^,^2^. Although the rates for new NHL cases were relatively stable over the last 10 years and death rates had been falling on average by 2.5% each year over 2003–2012, data from the National Cancer Institute had shown that there were an estimated 549, 625 people living with NHL in the United States in 2012 (http://seer.cancer.gov/statfacts/html/nhl.html). It has been estimated that there will be 72,850 new cases (40,170 males and 32,410 females) and 20,150 deaths (11,520 males and 8630 females) of NHL in the United States in 2016, accounting for 4.3% of all new cancer cases and 3.4% of all cancer deaths, respectively^3^. Moreover, the situation in China is also tough. As shown in a the latest report from China, new cases of lymphoma were estimated to be 88,200 (53,000 males and 35,200 females), and deaths were estimated to be 52,100 (32,700 males and 19,400 females) in 2015^4^. Although many aggressive lymphomas were very responsive to therapy such that they could be controlled for prolonged periods with evident improvement from treatment, death still occurred prematurely resulting from transformation into a more aggressive lymphoma or the side effects of therapy^5^. It has been reported that indolent lymphomas of different types could transform into high-grade malignancies, and this change occurred in up to 40% of patients and led to death^6^.

In fact, the incidence of NHL has nearly doubled in western countries since the 1970s, although no further increase has been found since the mid-1990s^7^. The etiologies of most NHL continued to be largely unknown, and this increase in NHL was partially explained by changes in diagnostic patterns, increased rates of HIV infection, use of immunosuppressive drugs, occupations or occupational and environmental risk factors (polychlorinated biphenyls, pesticides, solvents, hair dyes), and inherited genetic variations^8^,^9^. In recent decades, growing evidence has suggested that organochlorine compounds play an important role in the occurrence and development of NHL^10^.

Organochlorine pesticides (OCPs), one of the most abundant of the persistent organic pollutants (POPs), have been used extensively in agriculture as insecticides and for malaria prevention worldwide since the 1940s. Their properties, including persistence, long half-lives, high lipophilicity and long-range transport, and potential toxicity, have enabled them to be bioaccumulated in human tissue through the food chain and pose adverse impacts on human health^11^,^12^. Additionally, some OCPs, such as dichlorodiphenyltrichloroethane (DDT), hexachlorocyclohexane (HCH), hexachlorobenzene (HCB), chlordane, and heptachlor, had been found to be carcinogenic and thus were classified as possibly carcinogenic to humans (Group 2B) by the International Agency for Research on Cancer (IARC)^13^. However, though OCPs had been banned for more than 30 years in many countries due to their increasing adverse effects, OCPs could still be detected in soil, water, air, and foods, and eventually in human blood, adipose tissue, and breast milk^14^,^15^,^16^.

A number of studies have recently associated the risk of NHL with some environmental pollutants, including OCPs, with inconsistent results. For instance, Spinelli *et al*.^17^ reported that OCPs (β-HCH, p,p′-DDE, HCB, mirex, oxychlordane, and trans-nonachlor) had a significantly statistical association with increased risk of NHL in a population-based case-control study in British Columbia, Canada^17^. Quintana *et al*.^18^ conducted a nested case-control study from the US Environmental Protection Agency National Human Adipose Tissue Survey and concluded that adipose tissue levels of OCPs (heptachlor epoxide, oxychlordane, dieldrin, p,p′-DDE, and β-BHC) were associated with increased risk of NHL in a single pesticide analysis among a general population^18^. In contrast, some studies had failed to demonstrate the association between the exposure to OCPs and NHL risk^7^,^19^,^20^.

In the present study, we performed a meta-analysis to quantitatively evaluate whether the exposure to OCPs (mainly DDT, DDE, HCB, HCH, and chlordane) was associated with the risk of NHL with large sample sizes on the basis of published epidemiological evidence.

## Results

### Literature search and study characteristics

**Figure 1:**
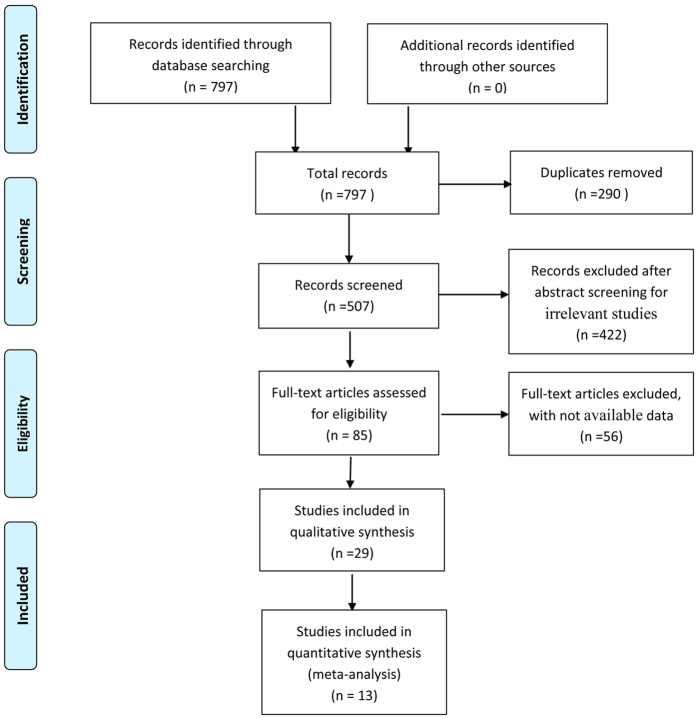
Flow diagram of literature search and study selection progression.

The results of literature research and selection progression were shown in Fig. 1. We identified 797 studies, of which 296 articles were from PubMed, 405 from Web of Science, and 96 from Embase prior to June 2015. After the exclusion of duplicate records and studies based on our inclusion criteria, there were 85 articles that were potentially relevant for this meta-analysis. Furthermore, after evaluating the full text of 85 articles in detail for further eligibility, 13 articles (6 nested case-control, 1 case-cohort, and 6 case-control studies) were ultimately included in our meta-analysis, with 37 independent studies stratified by objects. General characteristics of the included studies had been summarized in Supplementary Table S1 and Table S2. The meta-analysis consisted of 6582 cases and 9126 controls. Among all of the included studies, 21 studies were conducted in North America and 16 in Europe. According to the type of samples, the OCPs concentrations of 14 studies were measured in adipose tissue, 20 in blood, and 3 in adipose tissue and blood. Seventeen were published before 2005 (including 2005), while the others were published after 2005. The results of the quality assessment (score 0–9) of all included studies had been reported in the Supplementary Table S3 and Table S4 with a range in scores of 4–8, except one article with a score of 2.

### Association between OCPs and NHL risk

**Figure 2:**
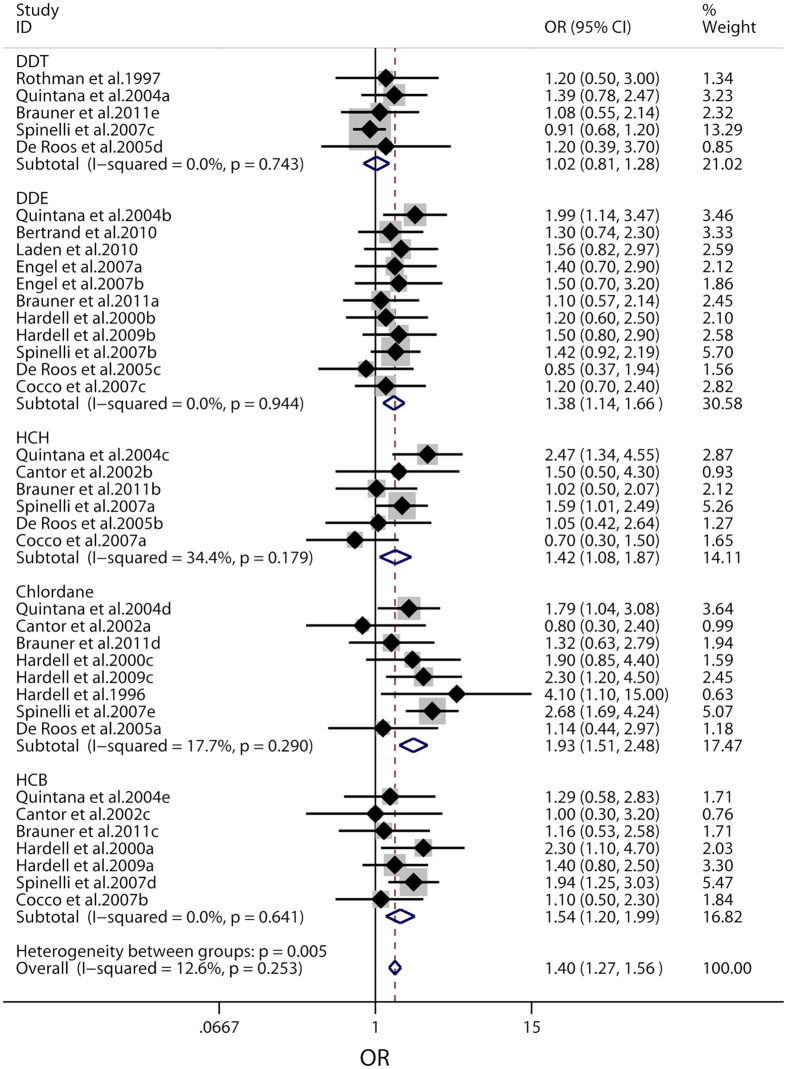
Forest plot of pooled ORs with 95% CIs for the association of organochlorine pesticides and non-Hodgkin lymphoma risk. The solid diamonds and horizontal lines correspond to study-specific ORs and 95% CIs. The gray areas reflect the study-specific weight. The hollow diamonds represent the pooled OR and 95% CI of each OCPs and the summary OR. The vertical solid lines show the OR of 1 and the vertical dashed lines indicate the corresponding pooled ORs.

Based on the results from the Cochrane Q-test with a P-value of 0.253 for heterogeneity, the fixed-effect model was applied to evaluate the risk of NHL related to OCPs. As shown in Fig. 2, the OCPs levels had statistically significant correlation with NHL risk (OR = 1.40, 95% CI 1.27 to 1.56), with the P-value for heterogeneity (P_h_) of 0.253 and I^2^-value of 12.6%. As for associations of exposure to specific objects and the risk of NHL, 5 main classifications of OCPs, including DDT, DDE, HCH, chlordane, and HCB, were studied independently. According to our results, the exposure to DDE, HCH, chlordane, and HCB had been found to increase the risk of NHL (DDE, OR = 1.38, 95% CI 1.14 to 1.66, P_h_ = 0.944, I^2^ = 0.0%; HCH, OR = 1.42, 95% CI 1.08 to 1.87, P_h_ = 0.179, I^2^ = 34.4%; chlordane, OR = 1.93, 95% CI 1.51 to 2.48, P_h_ = 0.290, I^2^ = 17.7%; HCB, OR = 1.54, 95% CI 1.20 to 1.99, P_h_ = 0.641, I^2^ = 0.0%; respectively), while non-significant association was confirmed between DDT exposure and NHL risk (DDT, OR = 1.02, 95% CI 0.81 to 1.28, P_h_ = 0.743, I^2^ = 0.0%).

### Subgroup analyses

**Figure 3:**
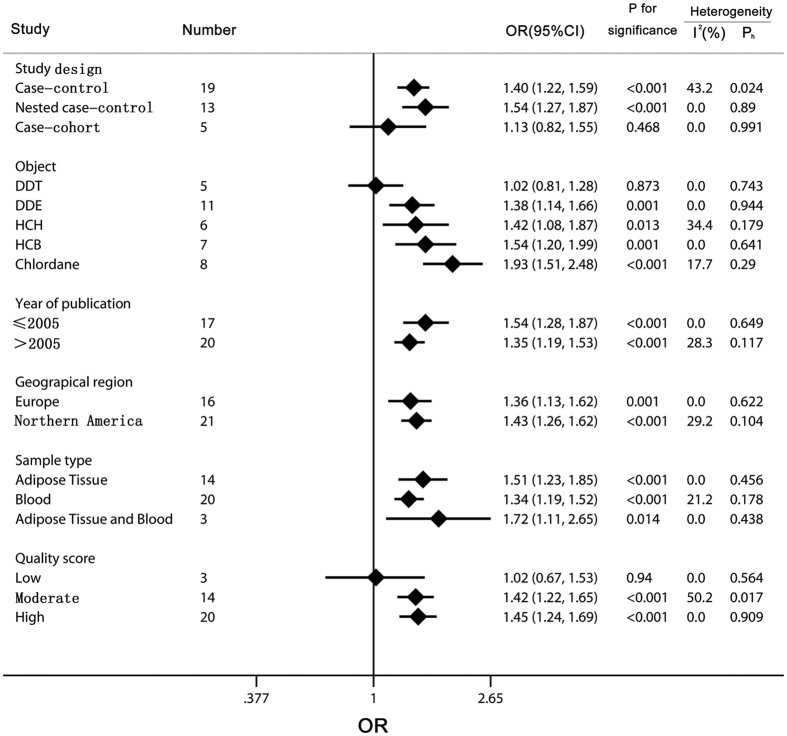
Subgroup analysis of organochlorine pesticides exposure in relation to non-Hodgkin lymphoma risk. The diamonds and horizontal lines represent the corresponding ORs and 95% CIs. The vertical lines show the OR of 1. Especially, for the year of publication, “≤2005” represents the studies published before 2005. As for geographical region, Europe includes Norway, Denmark, Sweden, Spain, France, and Germany, and America includes the United States and Canada. According to the 9-star Newcastle–Ottawa Scale, the scores are assigned as 0–3, 4–6, and 7–9 for low, moderate, and high quality of studies, respectively.

As shown in Fig. 3, subgroup analyses were conducted to explore potential sources of heterogeneity and examine various aspects of the studied association. We combined the ORs and 95% CIs from the subgroup analyses according to study design, object, year of publication, geographical region, sample type, quality score, whether adjusted by age or body mass index (BMI), and number of adjustments based on the general characteristics summary of the included studies. When the studies were stratified by the study design, the statistically significant increased risk for NHL was observed in both case-control and nested case-control studies (case-control, OR = 1.40, 95% CI 1.22 to 1.59, nested case-control, OR = 1.54, 95% CI 1.27 to 1.87), with a moderate heterogeneity in case-control studies (P_h_ = 0.024, I^2^ = 43.2%) and a low heterogeneity in nested case-control studies (P_h_ = 0.89, I^2^ = 0.0%), but the association was not found between OCPs and NHL risk in case-cohort studies (OR = 1.13, 95% CI 0.82 to 1.55, P_h_ = 0.991, I^2^ = 0.0%). When stratified by objects, DDE, HCH, chlordane, and HCB showed a significant association (DDE, OR = 1.38, 95% CI 1.14 to 1.66, HCH, OR = 1.42, 95% CI 1.08 to 1.87, chlordane, OR = 1.93, 95% CI 1.51 to 2.48, HCB, OR = 1.54, 95% CI 1.20 to 1.99) with NHL risk, while a non-significant association was suggested for DDT (OR = 1.02, 95% CI 0.81 to 1.28). According to year of publication and geographical region, significantly increased associations between OCPs levels and NHL risk were found in subgroups of year of publication (≤2005 or >2005) and geographical region (Europe or Northern America). Within the subgroup analyses of sample type, the results showed that the level of OCPs exposure determined from the types of mixed (adipose tissue and blood) and adipose tissue alone had a higher and significant risk for NHL (adipose tissue and blood, OR = 1.72, 95% CI 1.11 to 2.65, adipose tissue, OR = 1.51, 95% CI 1.23 to 1.85) than that from blood sample (OR = 1.34, 95% CI 1.19 to 1.52). In addition, the subgroup of quality score was examined, and there was significant association among the groups with moderate and high quality (moderate, OR = 1.42, 95% CI 1.22 to 1.65, high, OR = 1.45, 95% CI 1.24 to 1.69), but not with low quality (OR = 1.02, 95% CI 0.67 to 1.53).

**Figure 4:**
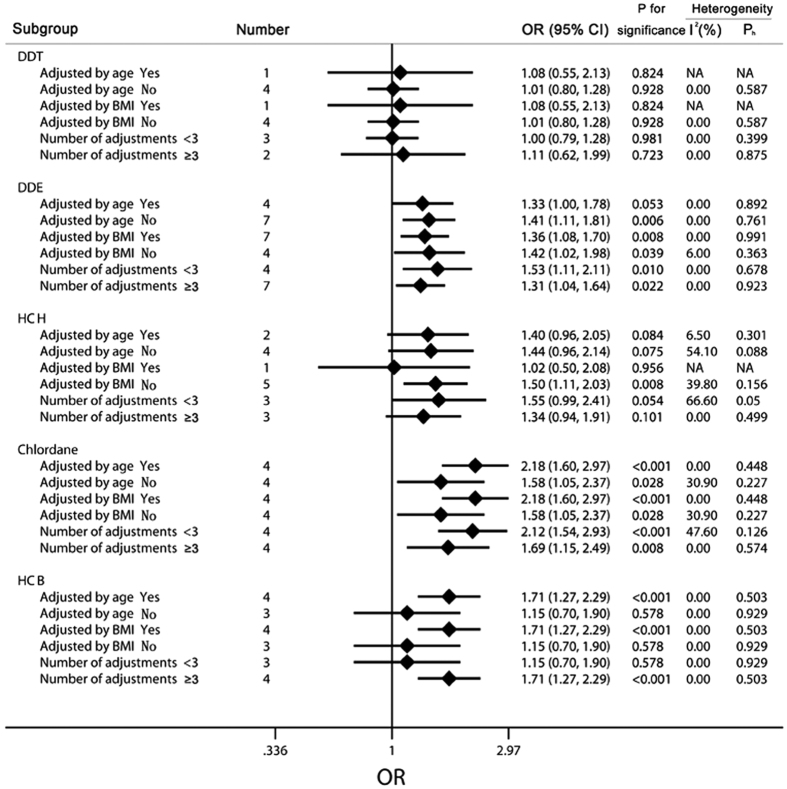
Subgroup analysis of each individual organochlorine pesticides. The diamonds and horizontal lines represent the corresponding ORs and 95% CIs. The vertical lines show the OR of 1. For the subgroup stratified by the number of adjustments, “≥3” indicates that the study should be adjusted for at least 3 of the confounding factors.

Meanwhile, to further evaluate the relationships among the exposures to the 5 main types of OCPs (DDT, DDE, HCH, chlordane, and HCB) and the risks for NHL, we conducted another subgroup analysis among these 5 objects by the group stratified by age, BMI, and number of adjustments, and detailed results had been shown in Fig. 4. Within the DDT group, none of our results suggested a significant association between the exposure to DDT and the risk of NHL (adjusted by age, OR = 1.08, 95% CI 0.55 to 2.13, non-adjusted by age, OR = 1.01, 95% CI 0.80 to 1.28; adjusted by BMI, OR = 1.08, 95% CI 0.55 to 2.13, non-adjusted by BMI, OR = 1.01, 95% CI 0.80 to 1.28; number of adjustments <3, OR = 1.00, 95% CI 0.79 to 1.28, number of adjustments ≥3, OR = 1.11, 95% CI 0.62 to 1.99). In contrast to the conclusions from the DDT group, there were significant relationships among the exposure of DDE and chlordane and NHL, even after the adjustment by age, BMI, and other adjustments (DDE adjusted by age, OR = 1.33, 95% CI 1.00 to 1.78, non-adjusted by age, OR = 1.41, 95% CI 1.11 to 1.81; adjusted by BMI, OR = 1.36, 95% CI 1.08 to 1.70, non-adjusted by BMI, OR = 1.42, 95% CI 1.02 to1.98; number of adjustments <3, OR = 1.53, 95% CI 1.11 to 2.11, number of adjustments ≥3, OR = 1.31, 95% CI 1.04 to 1.64; chlordane adjusted by age, OR = 2.18, 95% CI 1.60 to 2.97, non-adjusted by age, OR = 1.58, 95% CI 1.05 to 2.37; adjusted by BMI, OR = 2.18, 95% CI 1.60 to 2.97, non-adjusted by BMI, OR = 1.58, 95% CI 1.05 to 2.37; number of adjustments <3, OR = 2.12, 95% CI 1.54 to 2.93, number of adjustments ≥3, OR = 1.69, 95% CI 1.15 to 2.49). As for the subgroup analysis of the HCH group, only higher level of HCH was found to increase the risk of NHL when BMI was not adjusted (OR = 1.50, 95% CI 1.11 to 2.03). Besides, there were marginal significant associations observed among the groups, including those adjusted by age, non-adjusted by age, and with <3 adjustments (adjusted by age, OR = 1.40, 95% CI 0.96 to 2.05, non- adjusted by age, OR = 1.44, 95% CI 0.96 to 2.14, number of adjustments <3, OR = 1.55, 95% CI 0.99 to 2.41). When it came to the HCB group, elevated risk of NHL was found among individuals with higher HCB levels when the age, BMI or >3 adjustments (including 3) were adjusted (adjusted by age, OR = 1.71, 95% CI 1.27 to 2.29; adjusted by BMI, OR = 1.71, 95% CI 1.27 to 2.29; number of adjustments ≥3, OR = 1.71, 95% CI 1.27 to 2.29), while the non-adjusted groups and the group with <3 adjustments suggested that the association was non-significant (non-adjusted by age, OR = 1.15, 95% CI 0.70 to 1.90; non-adjusted by BMI, OR = 1.15, 95% CI 0.70 to 1.90; number of adjustments <3, OR = 1.15, 95% CI 0.70 to 1.90).

### Sensitivity analyses

**Figure 5:**
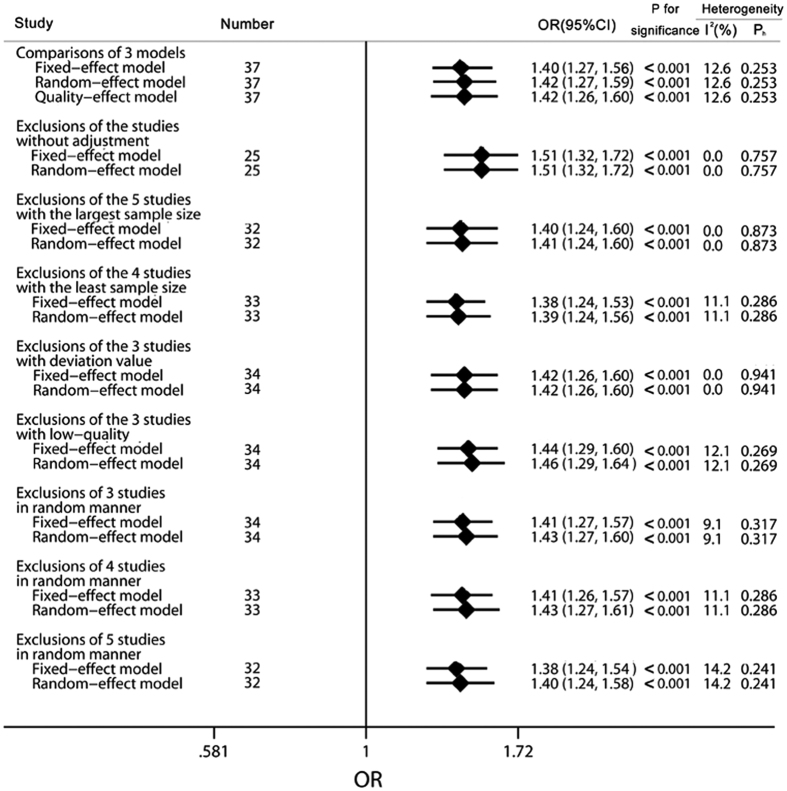
Results of sensitivity analysis on the relationship between organochlorine pesticides and non-Hodgkin lymphoma risk. The diamonds and horizontal lines represent the corresponding ORs and 95% CIs. The vertical lines show the OR of 1. “Studies without adjustment” includes Quintana *et al*.^18^, Cantor *et al*. 2002, Hardell *et al*.^41^, Spinelli *et al*. 2007c, and Cocco *et al*. 2007. “Studies with the largest sample size” includes Spinelli *et al*. 2007a, Spinelli *et al*. 2007b, Spinelli *et al*. 2007c, Spinelli *et al*. 2007d, and Spinelli *et al*. 2007e. “Studies with the least sample size” includes Hardell *et al*.^41^, Hardell *et al*. 2000a, Hardell *et al*. 2000b, and Hardell *et al*. 2000c. “Studies with deviation value” includes Quintana *et al*. 2004c, Spinelli *et al*. 2007c, and Spinelli *et al*. 2007e. “Studies with low-quality” includes Cocco *et al*. 2007a, Cocco *et al*. 2007b and Cocco *et al*. 2007c.

In order to evaluate the robustness of our conclusions about the exposure levels of OCPs on NHL risk, sensitivity analyses were conducted by different manners of exclusions as shown in Fig. 5. Similar results were observed when the outcomes of fixed-effect, random-effect, and quality-effect models (Supplementary Figure S1) were compared to each other (fixed-effect model, OR = 1.40, 95% CI 1.27 to 1.56; random-effect model, OR = 1.42, 95% CI 1.27 to 1.59; quality-effect model, OR = 1.42, 95% CI 1.26 to 1.60) with the same heterogeneity of P_h_ = 0.253 and I^2^ = 12.6%. In particular, exclusion of the studies with non-adjustment, the largest sample size, the smallest sample size, deviation values, and low-quality scores was performed to assess reliability and stability based on fixed-effect and random-effect models, the results of which were consistent with the pooled OR of OCPs exposure levels on NHL risk. We also omitted 3, 4, and 5 studies in a random manner using both fixed-effect and random-effect models for further study. Leave-one-out analysis was conducted by excluding one study in turn, and the pooled ORs and 95% CIs did not change dramatically as shown in Supplementary Figure S2. In general, findings from sensitivity analyses did not substantially alter the results of the overall pooled OR, thus the conclusions of our study were reliable and stable.

### Publication bias

**Figure 6:**
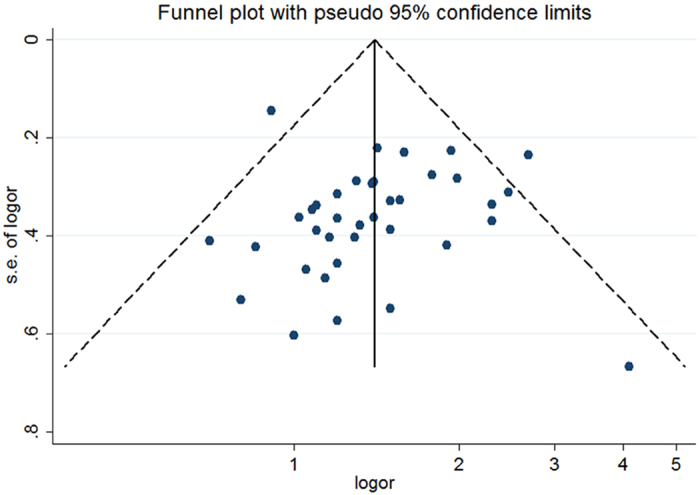
Funnel plot for studies of publication bias on the relationship between organochlorine pesticides and non-Hodgkin lymphoma risk. The horizontal line represents the summary effect estimates, and the dotted lines are pseudo 95% CIs.

Figure 6 showed little asymmetry of the visual inspection of the funnel plot. In addition, the Egger test (P = 0.853) did not suggest significant evidence of publication bias and the P value of the Begg test (P = 0.048) was marginally significant. Thus, publication bias was assessed to be ignorable in our analysis.

## Discussion

The association of OCPs exposure and NHL risk was first suggested by a few epidemiological studies among farmers who were supposed to have a high possibility of exposure to OCPs during their agricultural work^21^,^22^. However, due to the largely unexplained etiology of NHL, the biological plausibility and the potential mechanisms of the association between OCPs and NHL were difficult to ascertain. On one hand, since some OCPs, including DDT, HCH, HCB, chlordane, and heptachlor, had been classified as possible carcinogens to humans (Group 2B) by the IARC, their carcinogenicity had been speculated to result in the occurrence and development of NHL. On the other hand, immune system dysfunction has been found to be the potential mechanism for the development of NHL^23^,^24^,^25^. The dysregulation or suppression of T-cell function and immunosuppression were mainly involved in the dysfunction of the immune system to induce NHL tumors. When the T-cell function was dysregulated or suppressed, the Epstein-Barr virus–driven B-cell proliferation and transformation would be motivated. Subsequently, the physiologically occurring DNA double-strand breaks would lead to aberrant chromosomal translocations. As a result, chromosomal translocations had been observed in up to 90% of NHL cases^25^,^26^,^27^. Besides, either primary or genetic disorders of immune dysfunction and acquired states of severe immunosuppression (HIV/AIDS and organ transplantation) constituted strong and well-established risk factors for NHL, and it had been suggested that minor degrees of immunodeficiency also might mediate the development of lymphoma^25^,^26^. Organochlorine pesticides, particularly DDT, DDE, HCH, HCB, and chlordane, were widely used worldwide in the past few decades. Evidence has indicated that OCP exposures had adverse effects on the human immune system due to their immunotoxicity^28^,^29^. In direct immunotoxicity, OCPs could disturb the immune system during development, expansion, and survival signaling in immune cells, which might hinder the development and viability of immunocytes involved in the main pathways, such as induction of oxidative stress, mitochondrial dysfunction, endoplasmic reticulum stress, disruption of the ubiquitin protease system or autophagy, and inhibition of enzymes with esterase activity^30^. In animal experiments, pesticide-induced immunosuppression has been found that utero exposure to chlordane led to suppression of T lymphocyte function in mice. A previous study reported that cis-nonachlor, trans-nonachlor, and chlordane affected the immune system of Sprague–Dawley rats^31^. In human studies, the association between pesticide exposure and immune suppression was reported among children in Moldova. Some immunological abnormalities, including a lower percentage of natural killer cells, lower counts on mitogen stimulation assays, and a suggestion of a decrease in CD4 and an increase in CD8 cells, were observed among residents living in proximity to a pesticide dump site^23^. In addition, OCPs as environmental endocrine disruptors could profoundly disturb both homeostasis and function of the immune system through dysregulation of gonadotropin-releasing hormone (GnRH) biosynthesis in the hypothalamus gland^30^. Thus, it could be assumed that the carcinogenicity and the impairment of the immune system induced by OCPs exposure could be the potential mechanism of NHL tumor development.

In spite of the considerable effects from the epidemiological studies, the association between OCPs exposure and NHL risk has remained unclear. Thus, to evaluate the association between OCPs exposure and NHL risk, we conducted the present meta-analysis to pool the results from 13 relevant studies. As for the overall association, a significantly increased association between OCPs exposure and NHL risk was found (OCPs Summary OR = 1.40, 95% CI 1.27to 1.56), with a low heterogeneity of the included studies (P_h_ = 0.253, I^2^ = 12.6%). Besides, specific OCPs had been studied independently. The results had suggested that body burden levels of OCPs, including DDE, HCH, chlordane, and HCB, were significantly associated with increased risk of NHL (DDE, OR = 1.38, 95% CI 1.14 to 1.66; HCH, OR = 1.42, 95% CI 1.08 to 1.87; chlordane, OR = 1.93, 95% CI 1.51 to 2.48 ; HCB, OR = 1.54, 95% CI 1.20 to 1.99), while DDT exposure showed no evidence for a significant link with NHL risk (DDT, OR = 1.02, 95% CI 0.81 to 1.28).

Meta-analysis has the advantage of clarifying the association with a larger power than that from separate studies. However, potential bias should be considered in the meta-analysis when pooling results. Within the group by study design, the case-cohort studies group had a slightly elevated but not statistically significant risk for NHL (OR = 1.13, 95% CI 0.82 to 1.55), while the nested case-control studies group had a larger risk than the case-control studies group, reaching a statistically significant level (nested case-control, OR = 1.54, 95% CI 1.27 to 1.87; case-control, OR = 1.40, 95% CI 1.22 to 1.59). The exposure data in nested case-control and case-cohort designs are obtained on a subset of the full cohort, which are the most common approaches for reducing the costs of exposure assessment in prospective epidemiological studies. Furthermore, the cohort design was seen as a more valid method than the case-control design, which was more prone to potential biases related to control selection and recall, thus cohort results are more reliable^32^. Notably, in our analysis, there was no heterogeneity among the group of nested case-control studies and case-cohort studies, which implied that the present method to pool the results from these studies was reasonable. Thus, the conclusions from the group of nested case-control studies and case-cohort studies should be more credible. However, possibly due to the limited number of relevant studies, the conclusion was not significant in the case-cohort studies group. Therefore, caution should be taken when implicating the results from this analysis. Meanwhile, in addition to the analyses on study design, selection and investigation bias were also considered using the Newcastle-Ottawa Scale (NOS). The quality score of our included studies showed that the overall quality of nested case-control studies was significantly higher than that of case-control studies as a result of the rigorous research design, with assessed selection bias. It was reported that risk estimates from the studies with low quality might be overestimated than those from studies of high quality. Thus, the conclusions from nested case-control studies were reliable and accurate. Since year of publication was a vital factor that reflected the quality, we also conducted an analysis according to the year of publication. Generally speaking, recent studies were thought to possess a more rigorous design than those published in the past, thus results from recent studies were more rigorous and reliable. Clearly, in our analysis, the pooled ORs of the included studies published after 2005 (OR = 1.35, 95% CI 1.19 to 1.53) showed a lower risk of NHL than those published during or before 2005 (OR = 1.54, 95% CI 1.28 to 1.87). It could be supposed that the relationship might be overestimated among the studies published during or before 2005 due to the poorer design. However, the conclusions remain unchanged and statistically significant.

NHL was more common in developed areas, with the highest incidence rates found in Australia, Western and Northern Europe, and Northern America. The lowest NHL rates were found in Asia and Eastern Europe, except some special histological subtypes. Exactly, the geographical region of the studies in our meta-analysis mainly included Europe (Norway, Denmark, Sweden, Spain, France, Germany) and Northern America (the United States, Canada), with a distinct increased risk of NHL in Northern America (OR = 1.43, 95% CI 1.26 to 1.62) and Europe (OR = 1.36, 95% CI 1.13 to 1.62). However, we should note that studies from Asia and Africa were lacking in our meta-analysis, and these findings need to be interpreted with caution. Further comprehensive studies should be conducted in Asia and less developed countries to further determine the geographical difference in risk of NHL.

OCPs were reported to possess high lipophilicity, thus they could be bioaccumulated in human tissue through the food chain and pose a threat to human health^11^,^12^. Theoretically, OCPs were likely to concentrate in the sample type of adipose tissue that contained higher fat content, and lipid weight was likely to be one source of heterogeneity. Although we found no evidence that sample type might affect results, our results exhibited a significantly higher NHL risk in adipose tissue than in blood (adipose tissue, OR = 1.51, 95% CI 1.23 to 1.85; blood, OR = 1.34, 95% CI 1.19 to 1.52). Additionally, for the higher I^2^-value results from the blood subgroup than from adipose tissue, we believed that different definitions and methods of detection of total lipids might be potential reasons of heterogeneity, and the difference between serum and plasma might had affected the I^2^ test results in part. As for the group of mixed with adipose tissue and blood, it was not enough to examine our conclusions due to the limited studies.

In addition to the difference among the group by sample type, high lipophilicity of OCPs was hypothesized to lead to an increased risk among individuals with higher BMI. A report in 2014 evaluated the association between pre-diagnostic obesity and survival for patients with NHL, with the conclusions implying that pre-diagnostic obesity might be related to poorer overall and NHL-specific survival in patients with NHL^33^. A large population-based case-control study by Skibola *et al*. between 1988 and 1995 showed a positive correlation between increased BMI and the risk of NHL, especially diffuse large cell lymphoma and follicular lymphoma^34^. Meanwhile, leptin had been suggested to be a mediator in the pathogenesis of this disease^34^. Another case-control study was carried out in England by Willett *et al*. and raised the possibility that obesity was positively associated with an increased risk of NHL (OR = 1.5, 95% CI 1.1 to 2.1)^35^. In addition, BMI was found to be significantly positively associated with risk of diffuse large B-cell lymphoma in a meta-analysis of prospective studies published in 2011^36^. Owing to the major confounding effect of BMI, the adjustment of BMI should be conducted to obtain reliable conclusions. In our findings, DDE, chlordane, and HCB adjusted by BMI showed a statistically significant correlation with risk of NHL, in which the DDE and HCH groups that were non-adjusted by BMI had indicated a higher risk than those adjusted by BMI (DDE adjusted by BMI, OR = 1.36, 95% CI 1.08 to 1.70; DDE non-adjusted by BMI, OR = 1.42, 95% CI 1.02 to 1.98; HCH adjusted by BMI, OR = 1.05, 95% CI 0.50 to 2.08; HCH non-adjusted by BMI, OR = 1.50, 95% CI 1.11 to 2.03). Consequently, this suggested that BMI had a potential effect on the increased risk of NHL and the positive relationship of exposure to OCPs and NHL risk might be more truly expressed in groups when adjusted by BMI, which further confirmed the reliability and robustness of our results.

Age was also suggested to be a major confounder in the association between the exposure to OCPs and the risk of NHL. For one side, OCPs were reported to possess long half-lives, enabling them to maintain a stable structure inside the human body and adversely affect human health for a long period. Under these circumstances, the exposure level of OCPs could increase with age, thus older individuals could have a higher level of OCPs exposure. For the other side, age was an essential factor in the occurrence and development of a variety of cancers, including NHL tumors. It had been suggested that age might play an essential role in the incidence of NHL, and the overall incidence of NHL increases steadily with age at diagnosis^37^. Zheng *et al*. observed a higher incidence rate in older age groups than in younger age groups for all birth cohorts examined, and recent cohorts had a higher incidence rate than earlier cohorts, especially among the older age groups in both men and women based on all NHL cases reported to the Connecticut Tumor Registry between 1935 and 1988^37^. Another study on incidence rates and trends of NHL from 1995 to 2004 was performed in Alexandria, Egypt, by Abdel-Fattah & Yassine, and consequently doubled incidence was also found in the older age group^38^. Therefore, it was considerably necessary to adjust for age to explore the reliable relationship between OCPs exposure and NHL risk based on a broad range of ages in our included studies. Our results of DDE and HCH in detailed subgroup analyses supported the opinion that age is a potential confounding factor for NHL risk (DDE adjusted by age OR = 1.33, 95% CI 1.00 to 1.78, DDE non-adjusted OR = 1.41, 95% CI 1.11 to 1.81; HCH adjusted by age OR = 1.40, 95% CI 0.96 to 2.05, HCH non-adjusted OR = 1.44, 95% CI 0.96 to 2.14). Our results were in correspondence with the previously reported conclusions that age would magnify the association between OCP exposure and NHL risk.

Apart from the potential confounding effects of age and BMI, analysis on the numbers of adjustments was conducted. The total identified studies were stratified by the numbers of adjustments. Consequently, the results revealed that the groups with 3 or more adjustments (DDE, OR = 1.31, 95% CI 1.04 to 1.64; HCH, OR = 1.34, 95% CI 0.94 to 1.91, chlordane, OR = 1.69, 95% CI 1.15 to 2.49) showed a lower risk than the groups with less than 3 adjustments (DDE, OR = 1.53, 95% CI 1.11 to 2.11; HCH, OR = 1.55, 95% CI 0.99 to 2.41, chlordane, OR = 2.12, 95% CI 1.54 to 2.93). Results with more adjustments would be more likely to express the true association. Nonetheless, these findings confirmed the reliability of our results, which remained consistent even after the adjustments of major confounding factors.

Several limitations and uncertainties of our meta-analysis should be acknowledged. Firstly, publication bias and selection bias might be inevitable due to the inclusion of only published studies and with the exclusion of non-English studies. Secondly, though most studies gave a sufficient exposure gradient in the NHL risk assessment, tertile, quartile, and quintile were used for the OR estimate via setting the lowest quantile as the reference. Therefore, the exposure measurement was different across the studies, which might be a potential source of heterogeneity in this analysis. Thirdly, the exposure to OCPs (i.e., DDE, DDT, HCH, HCB, and chlordane) with estrogenic properties might pose an increased NHL risk as a result of the interactions among these chemicals. Finally, since various subtypes of NHL had different characteristics and possibly diverse etiologies, the associations with overall NHL, rather than with subtypes of NHL, would lead to uncertain problems, thus the results should be interpreted with caution.

In conclusion, the findings of our meta-analysis yielded a statistically significant association between exposure to OCPs and elevated risk of NHL. Subgroup analyses of DDE, chlordane, and HCB further confirmed the reliability of our results, which remained consistent even after adjustments of the major confounding factors (age and BMI). Future research with larger sample sizes is warranted to confirm these findings and to determine the likely biological mechanism.

## Materials and Methods

### Search identification

In this meta-analysis, the literatures were identified by a search on PubMed, Web of Science and Embase from the period 1986 to June 2015 for observational studies in relation to OCPs and NHL. Various combinations of the following keywords were used in our search strategy: “organochlorine”, “pesticide”, “DDT”, “DDE”, “HCB”, “HCH”, “chlordane” and “non-Hodgkin lymphoma”. Moreover, the reference lists of the selected articles were also reviewed for potentially eligible studies. The identified articles were limited to studies published in English. This meta-analysis had been conducted according to the PRISMA checklists and followed the guideline to report the results^39^.

### Inclusion criteria

Studies were considered eligible in the meta-analysis if they satisfied the following criteria: (1) the study design was observational, (2) the exposure of interest was DDT, DDE, HCB, HCH, chlordane, (3) the measurement of the exposure of interest should be conducted in body fluids or tissues, not using questionnaires to review the exposure history, (4) the outcome of interest was NHL, (5) the study examined the association between DDT/DDE/HCB/HCH/chlordane exposure and NHL, and (6) the study should include reported ORs or relative risk (RR) with 95% CIs or provided sufficient information to extrapolate relevant values. Meanwhile, we excluded the studies that did not report original results, such as reviews, editorials, comments, correspondence, letters, meta-analyses, and meeting abstracts. We identified 797 studies through the systematic literature search, and finally 13 studies (6 nested case-control, 1 case-cohort, and 6 case–control) were included in this meta-analysis.

### Data extraction

Data extraction was performed independently and reviewed by 2 reviewers (D.L. and T.Z.), and the results of this data extraction were compared between the 2 reviewers, and consensus was obtained before the meta-analysis. The reviewers extracted the following information for each study: first author name, publication year, country of study, type of study design, number of cases or controls, type of OCPs, sample type, gender, age of cases and controls, OR and corresponding 95% CI, and the confounding factors used for the adjustment of ORs. The ORs with more adjusted confounders were chosen when studies had different models for the calculation of estimated risks.

### Quality assessment

According to the 9-star Newcastle–Ottawa Scale (NOS)^40^, the quality of all of the included studies underwent an independent assessment modified by 2 authors separately, which included selection (0–4 points), comparability (0–2 points), and exposure (0–3 points) for case-control, nested case-control, and case-cohort studies to assess the quality of these selected articles (Supplementary Table S3 and S4). The articles with higher scores were considered to be of better quality. Disagreements in quality assessment were resolved by discussions among the authors. The scores were assigned to 0–3, 4–6, and 7–9 for low, moderate, and high quality of studies, respectively. A study received 1 point if it met the established criteria, otherwise it received 0 points.

### Statistical analysis

Among the 13 identified studies, 4 studies^7^,^41^,^42^,^43^ included only one objects, the rest of the selected studies^8^,^17^,^18^,^20^,^44^,^45^,^46^,^47^,^48^ contained two or more target analytes. In this meta-analysis, all results were stratified by target analytes and treated as separate studies. Consequently, this meta-analysis included 37 independent reports in total. It was previously reported that OR, hazard ratio (HR), and RR would provide similar risk assessments when the outcome was rare^49^. Therefore, in this meta-analysis, we chose the published ORs and 95% CIs as the common effect size and transformed HRs or RRs into ORs if ORs were not available. If the crude and adjusted ORs were both shown in the study, we used the adjusted ORs. If the RR was reported instead of the OR, we used the RR instead.

Heterogeneity among the results of these studies was estimated using the Cochran Q test and I^2^ statistic^50^. Fixed-effect model was used to summarize study estimates comparing extreme categories (highest category vs. reference category and the ones with the most adjusted variables) of OCPs in each individual study when no evidence of heterogeneity (P > 0.1) was suggested by the Cochrane Q-test, otherwise random-effect model was applied^51^. Meanwhile, I^2^ values of 25%, 50%, and 75% as low, moderate, and high degrees of heterogeneity, respectively, were used to assess heterogeneity^50^. Besides, quality-effect model based on quality scores was used, and the results were compared with those from the random-effect and fixed-effect models.

Subgroup analyses according to study design, object, year of publication, geographical region, sample type, quality score, and adjustments were conducted with the hope to evaluate the potential sources of heterogeneity and to investigate the detailed aspects of the association between OCPs levels and the risk of NHL.

Sensitivity analyses were conducted to explore the effects of a specific study on the pooled OR. In addition to the Begg’s test^52^ and Egger’s test^53^, we also introduced a visual inspection of funnel plot showing the natural logarithm of the estimate of OR (logor) versus the standard error of logor to assess the potential for publication bias. The overall risk of NHL caused by exposure to OCPs was evaluated based on a fixed-effects model and was illustrated by forest plots.

All statistical analyses were performed using the Stata version 12.0 software (StataCorp LP, College Station, TX), other than quality-effect modeling (applying MetaXL version 2.2 software [EpiGear International Pty. Ltd., Queensland, Australia]). All statistical tests were two-tailed, and a P-value of <0.05 was considered statistically significant.

## Additional Information

**How to cite this article**: Luo, D. *et al*. Exposure to organochlorine pesticides and non-Hodgkin lymphoma: a meta-analysis of observational studies. *Sci. Rep.* **6**, 25768; doi: 10.1038/srep25768 (2016).

## Supplementary Material

Supplementary Information
