# Supplementary material for: Exposure to organochlorine pesticides and non-Hodgkin lymphoma: a meta-analysis of observational studies
Source: Sci Rep. 2016 May 17;6:25768. doi: 10.1038/srep25768 (PMC4869027; doi:10.1038/srep25768)
Supplement: Supplementary Information [file srep25768-s1.pdf]

## Supplementary Information

# Exposure to organochlorine pesticides and non-Hodgkin lymphoma: a meta-analysis of observational studies

Dan Luo[1], Tingting Zhou[1], Yun Tao[1], Yaqian Feng[1], Xiaoli Shen[1] & Surong Mei[1, *]

[1]State Key Laboratory of Environment Health (Incubation), Key Laboratory of Environment and Health, Ministry of Education, Key Laboratory of Environment and Health (Wuhan), Ministry of Environmental Protection, School of Public Health, Tongji Medical College, Huazhong University of Science and Technology, #13 Hangkong Road, Wuhan, Hubei, 430030, China

*Corresponding author:

Surong Mei; E-mail: surongmei@hust.edu.cn

Address: School of Public Health, Tongji Medical College, Huazhong University of Science and Technology, #13 Hangkong Road, Wuhan, Hubei, 430030, China;

Tel: +86(27)-83657849；

Fax: +86(27)-83657765.

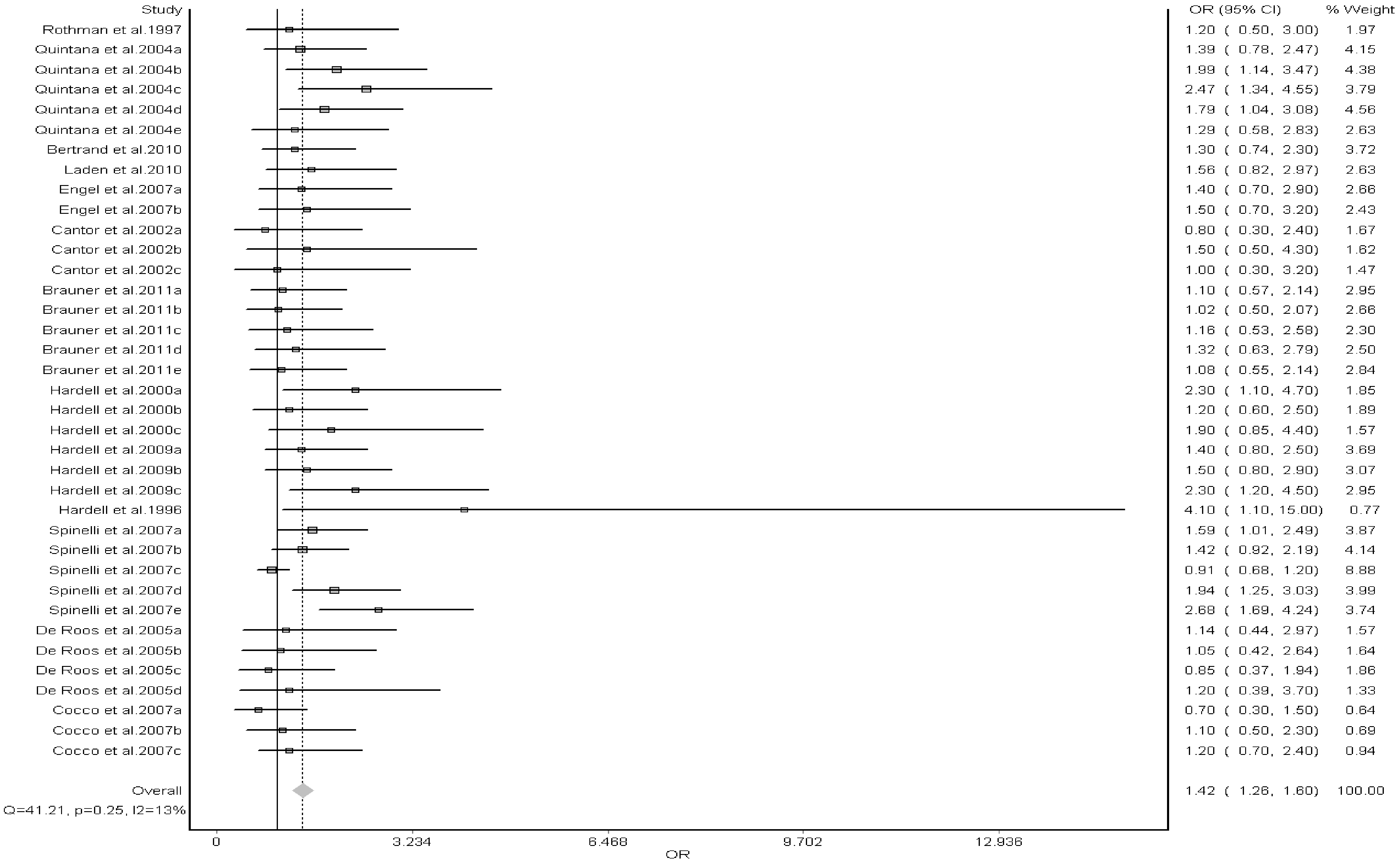


**Supplementary Figure S1. Pooled ORs with 95% CIs for the association of organochlorine pesticides and non-Hodgkin lymphoma risk based on quality-effect model.** The solid diamonds and horizontal lines correspond to the study-specific ORs and 95% CIs. The gray areas reflect the study-specific weight. The hollow diamonds represent the pooled OR and 95% CI of each OCPs and the summary OR. The vertical solid lines show the OR of 1 and the vertical dashed lines indicate the corresponding pooled ORs.

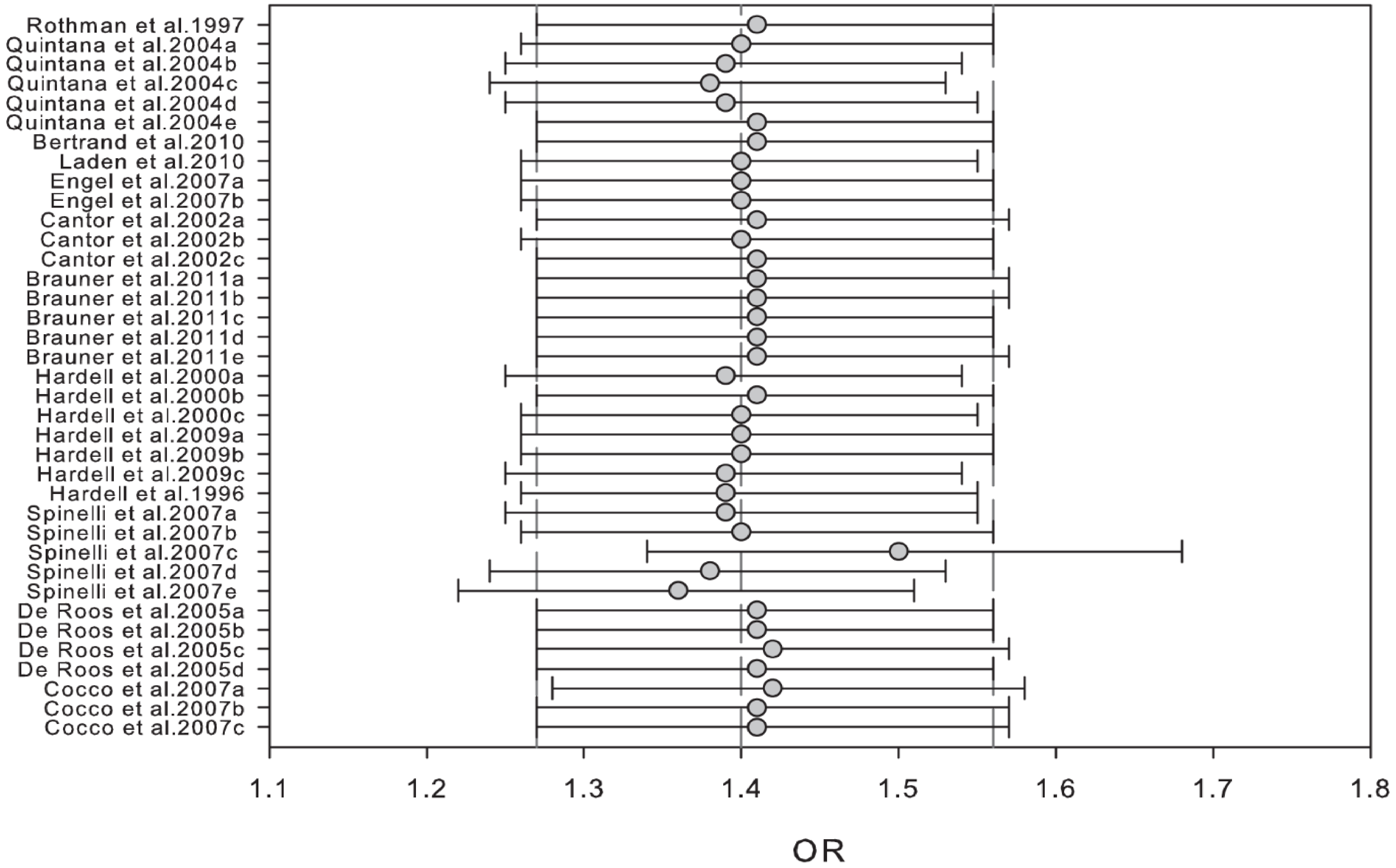


**Supplementary Figure S2. Results of sensitivity analysis using leave-one-out method.** The gray circles and the horizontal lines represent the pooled ORs and 95% CIs after omitting studies in turn.

**Supplementary Table S1. Characteristics of nested case-control studies and case-cohort studies relating organochlorine pesticides and non-Hodgkin lymphoma**

| Reference | Country | Cases/Controls | Objects[a] | Sample type | Gender[b] | Age（cases/controls）[c] | Unit | OR or RR(95% CI)[d] | Adjustments |
|---|---|---|---|---|---|---|---|---|---|
| **Nested case-control** | | | | | | | | | |
| Rothman et al.1997 | USA | 74/147 | DDT | Serum | F/M | 52.9/52.5（mean） | ng/g lipid | 1.2(0.50-3.00) | DDT and PCB risk estimates adjusted for each other |
| Quintana et al.2004a | USA | 175/481 | DDT | Adipose tissue | F/M | 49.0 ±22.4/50.0 ±21.9 | μg/g lipid | 1.39(0.78-2.47) | Crude |
| Quintana et al.2004b | USA | 175/481 | DDE | Adipose tissue | F/M | 49.0 ±22.4/50.0 ±21.9 | μg/g lipid | 1.99(1.14-3.47) | Crude |
| Quintana et al.2004c | USA | 175/481 | HCH | Adipose tissue | F/M | 49.0 ±22.4/50.0 ±21.9 | μg/g lipid | 2.47(1.34-4.55) | Crude |
| Quintana et al.2004d | USA | 175/481 | Chlordane | Adipose tissue | F/M | 49.0 ±22.4/50.0 ±21.9 | μg/g lipid | 1.79(1.04-3.08) | Crude |
| Quintana et al.2004e | USA | 175/481 | HCB | Adipose tissue | F/M | 49.0 ±22.4/50.0 ±21.9 | μg/g lipid | 1.29(0.58-2.83) | Crude |
| Bertrand et al.2010 | USA | 205/409 | DDE | Plasma | M | 40-84 | ng/g lipid | 1.3(0.74-2.30) | BMI,smoking,alcohol intake,height |
| Laden et al.2010 | USA | 145/290 | DDE | Plasma | F | 44-69 | ng/g lipid | 1.56(0.82-2.97) | Region,BMI,smoking status,parity,breastfeeding,height |
| Engel et al.2007a | Norway | 190/190 | DDE | Serum | F/M | 42.9 ±5.3/42.9 ±5.3 | ng/g lipid | 1.4(0.70-2.90) | BMI,smoking status |
| Engel et al.2007b | USA | 74/147 | DDE | Serum | F/M | 52.9±13.2/52.5±13.8 | ng/g lipid | 1.5(0.70-3.20) | Years of education,smoking status |
| Cantor et al.2002a | USA | 74/147 | Chlordane | Serum | F/M | NA | ng/g lipid | 0.8(0.30-2.40) | Crude |
| Cantor et al.2002b | USA | 74/147 | HCH | Serum | F/M | NA | ng/g lipid | 1.5(0.50-4.30) | Crude |
| Cantor et al.2002c | USA | 74/147 | HCB | Serum | F/M | NA | ng/g lipid | 1.0(0.30-3.20) | Crude |
| **Case-cohort** | | | | | | | | | |
| Brauner et al.2011a | Denmark | 239/245 | DDE | Adipose tissue | F/M | 64/56（median） | μg/kg lipid | 1.1(0.57-2.14) | Age,sex,BMI |
| Brauner et al.2011b | Denmark | 239/245 | HCH | Adipose tissue | F/M | 64/56（median） | μg/kg lipid | 1.02(0.50-2.07) | Age,sex,BMI |
| Brauner et al.2011c | Denmark | 239/245 | HCB | Adipose tissue | F/M | 64/56（median） | μg/kg lipid | 1.16(0.53-2.58) | Age,sex,BMI |
| Brauner et al.2011d | Denmark | 239/245 | Chlordane | Adipose tissue | F/M | 64/56（median） | μg/kg lipid | 1.32(0.63-2.79) | Age,sex,BMI |
| Brauner et al.2011e | Denmark | 239/245 | DDT | Adipose tissue | F/M | 64/56（median） | μg/kg lipid | 1.08(0.55-2.14) | Age,sex,BMI |

[a] DDT-dichlorodiphenyltrichloroethane, DDE-dichlorodiphenyldichloroethylene, HCH-hexachlorocyclohexane, HCB-hexachlorobezene.
[b] F means female, M means male, F/M means the study includes both female and male objects.
[c] NA means the data is not available.
[d] OR means odds ratio, and CI means confidence interval.

**Supplementary Table S2. Characteristics of case-control studies relating organochlorine pesticides and non-Hodgkin lymphoma**

| Reference | Country | Cases/Controls | Objects[a] | Sample type | Gender[b] | Age（cases/controls） | Unit | OR or RR (95% CI)[c] | Adjustments |
|---|---|---|---|---|---|---|---|---|---|
| Hardell et al.2000a | Sweden | 82/83 | HCB | Adipose tissue and blood | F/M | 62.5/62.3（mean） | ng/g lipid | 2.3(1.10-4.70) | Age,sex,BMI,specimen analysed（blood or adipose tissue） |
| Hardell et al.2000b | Sweden | 82/83 | DDE | Adipose tissue and blood | F/M | 62.5/62.3（mean） | ng/g lipid | 1.2(0.60-2.50) | Age,sex,BMI,specimen analysed（blood or adipose tissue） |
| Hardell et al.2000c | Sweden | 82/83 | Chlordane | Adipose tissue and blood | F/M | 62.5/62.3（mean） | ng/g lipid | 1.9(0.85-4.40) | Age,sex,BMI,specimen analysed（blood or adipose tissue） |
| Hardell et al.2009a | Sweden | 99/99 | HCB | Plasma | F/M | 55.9/56.4(mean) | ng/g lipid | 1.4(0.80-2.50) | Age,sex,BMI |
| Hardell et al.2009b | Sweden | 99/99 | DDE | Plasma | F/M | 55.9/56.4(mean) | ng/g lipid | 1.5(0.80-2.90) | Age,sex,BMI |
| Hardell et al.2009c | Sweden | 99/99 | Chlordane | Plasma | F/M | 55.9/56.4(mean) | ng/g lipid | 2.3(1.20-4.50) | Age,sex,BMI |
| Hardell et al.1996 | Sweden | 27/17 | Chlordane | Adipose tissue | F/M | 64.4/60.5（mean） | ng/g lipid | 4.1(1.10-150) | Crude |
| Spinelli et al.2007a | Canada | 422/460 | HCH | Plasma | F/M | 20-79 | ng/g lipid | 1.59(1.01-2.49) | Age,ethnicity,family history of NHL |
| Spinelli et al.2007b | Canada | 422/460 | DDE | Plasma | F/M | 20-79 | ng/g lipid | 1.42(0.92-2.19) | Age,BMI,ethnicity |
| Spinelli et al.2007c | Canada | 422/460 | DDT | Plasma | F/M | 20-79 | ng/g lipid | 0.91(0.68-1.20) | Crude |
| Spinelli et al.2007d | Canada | 422/460 | HCB | Plasma | F/M | 20-79 | ng/g lipid | 1.94(1.25-3.03) | Age,sex,BMI |
| Spinelli et al.2007e | Canada | 422/460 | Chlordane | Plasma | F/M | 20-79 | ng/g lipid | 2.68(1.69-4.24) | Age,BMI |
| De Roos et al.2005a | USA | 100/100 | Chlordane | Plasma | F/M | 20-74 | ng/g lipid | 1.14(0.44-2.97) | Sex,study site,birth date,date of blood draw |
| De Roos et al.2005b | USA | 100/100 | HCH | Plasma | F/M | 20-74 | ng/g lipid | 1.05(0.42-2.64) | Sex,study site,birth date,date of blood draw |
| De Roos et al.2005c | USA | 100/100 | DDE | Plasma | F/M | 20-74 | ng/g lipid | 0.85(0.37-1.94) | Sex,study site,birth date,date of blood draw |
| De Roos et al.2005d | USA | 100/100 | DDT | Plasma | F/M | 20-74 | ng/g lipid | 1.2(0.39-3.70) | Sex,study site,birth date,date of blood draw |
| Cocco et al.2007a | France,Germany ,Spain | 174/203 | HCH | Plasma | F/M | 58.0±15.6/54.7±15.6 | ng/mL | 0.7(0.30-1.50) | Crude |
| Cocco et al.2007b | France,Germany ,Spain | 174/203 | HCB | Plasma | F/M | 58.0±15.6/54.7±15.6 | ng/mL | 1.1(0.50-2.30) | Crude |
| Cocco et al.2007c | France,Germany ,Spain | 174/203 | DDE | Plasma | F/M | 58.0±15.6/54.7±15.6 | ng/mL | 1.2(0.70-2.40) | Crude |

[a] DDT-dichlorodiphenyltrichloroethane, DDE-dichlorodiphenyldichloroethylene, HCH-hexachlorocyclohexane, HCB-hexachlorobezene.
[b] F means female, M means male.
[c] OR means odds ratio, and CI means confidence interval

**Supplementary Table S3. Quality assessments of the nested case-control and case-cohort studies relating organochlorine pesticides and non-Hodgkin lymphoma**

| Study | Selection | | | | Comparability | | Exposure | | | Overall quality score |
|---|---|---|---|---|---|---|---|---|---|---|
| | 1 | 2 | 3 | 4 | 5A | 5B | 6 | 7 | 8 | |
| Rothman et al.1997 | 0 | 1 | 1 | 1 | 1 | 1 | 1 | 1 | 0 | 7 |
| Quintana et al.2004a | 1 | 1 | 1 | 1 | 1 | 1 | 1 | 1 | 0 | 8 |
| Quintana et al.2004b | 1 | 1 | 1 | 1 | 1 | 1 | 1 | 1 | 0 | 8 |
| Quintana et al.2004c | 1 | 1 | 1 | 1 | 1 | 1 | 1 | 1 | 0 | 8 |
| Quintana et al.2004d | 1 | 1 | 1 | 1 | 1 | 1 | 1 | 1 | 0 | 8 |
| Quintana et al.2004e | 1 | 1 | 1 | 1 | 1 | 1 | 1 | 1 | 0 | 8 |
| Bertrand et al.2010 | 0 | 1 | 1 | 1 | 1 | 1 | 1 | 1 | 0 | 7 |
| Laden et al.2010 | 0 | 1 | 1 | 1 | 1 | 1 | 1 | 0 | 0 | 6 |
| Engel et al.2007a | 0 | 1 | 1 | 1 | 1 | 1 | 1 | 1 | 0 | 7 |
| Engel et al.2007b | 0 | 1 | 1 | 1 | 1 | 1 | 1 | 1 | 0 | 7 |
| Cantor et al.2002a | 0 | 1 | 1 | 1 | 1 | 1 | 1 | 1 | 0 | 7 |
| Cantor et al.2002b | 0 | 1 | 1 | 1 | 1 | 1 | 1 | 1 | 0 | 7 |
| Cantor et al.2002c | 0 | 1 | 1 | 1 | 1 | 1 | 1 | 1 | 0 | 7 |
| Brauner et al.2011a | 0 | 1 | 1 | 1 | 1 | 1 | 1 | 1 | 0 | 7 |
| Brauner et al.2011b | 0 | 1 | 1 | 1 | 1 | 1 | 1 | 1 | 0 | 7 |
| Brauner et al.2011c | 0 | 1 | 1 | 1 | 1 | 1 | 1 | 1 | 0 | 7 |
| Brauner et al.2011d | 0 | 1 | 1 | 1 | 1 | 1 | 1 | 1 | 0 | 7 |
| Brauner et al.2011e | 0 | 1 | 1 | 1 | 1 | 1 | 1 | 1 | 0 | 7 |

*The study quality was assessed according to the Newcastle Ottawa Quality assessment scale for nested case-control studies. This scale awards a maximum of 9 points to each study: 4 for selection, 2 for comparability, and 3 for assessment of outcomes (for cohort study). 1 = "Yes", 0 = "No", "Unable to determine" or "Not available". For cohort studies, 1, indicates exposed cohort truly representative; 2, non-exposed cohort drawn from the same community; 3, ascertainment of exposure; 4, outcome of interest not present at start; 5A, cohorts comparable on basis of age; 5B, cohorts comparable on other factor(s); 6, quality of outcome assessment; 7, follow-up long enough for outcomes to occur; and 8, complete accounting for cohorts.

**Supplementary Table S4. Quality assessment of the case-control studies relating organochlorine pesticides and non-Hodgkin lymphoma**

| Study | Selection | | | | Comparability | | Exposure | | | Overall quality score |
|---|---|---|---|---|---|---|---|---|---|---|
| | 1 | 2 | 3 | 4 | 5A | 5B | 6 | 7 | 8 | |
| Hardell et al.2000a | 0 | 0 | 0 | 0 | 1 | 1 | 1 | 1 | 1 | 5 |
| Hardell et al.2000b | 0 | 0 | 0 | 0 | 1 | 1 | 1 | 1 | 1 | 5 |
| Hardell et al.2000c | 0 | 0 | 0 | 0 | 1 | 1 | 1 | 1 | 1 | 5 |
| Hardell et al.2009a | 1 | 1 | 1 | 0 | 1 | 1 | 1 | 1 | 0 | 7 |
| Hardell et al.2009b | 1 | 1 | 1 | 0 | 1 | 1 | 1 | 1 | 0 | 7 |
| Hardell et al.2009c | 1 | 1 | 1 | 0 | 1 | 1 | 1 | 1 | 0 | 7 |
| Hardell et al.1996 | 1 | 0 | 0 | 0 | 1 | 0 | 1 | 1 | 0 | 4 |
| Spinelli et al.2007a | 0 | 0 | 1 | 0 | 1 | 1 | 1 | 1 | 0 | 5 |
| Spinelli et al.2007b | 0 | 0 | 1 | 0 | 1 | 1 | 1 | 1 | 0 | 5 |
| Spinelli et al.2007c | 0 | 0 | 1 | 0 | 1 | 1 | 1 | 1 | 0 | 5 |
| Spinelli et al.2007d | 0 | 0 | 1 | 0 | 1 | 1 | 1 | 1 | 0 | 5 |
| Spinelli et al.2007e | 0 | 0 | 1 | 0 | 1 | 1 | 1 | 1 | 0 | 5 |
| De Roos et al.2005a | 1 | 0 | 1 | 0 | 1 | 1 | 1 | 1 | 0 | 6 |
| De Roos et al.2005b | 1 | 0 | 1 | 0 | 1 | 1 | 1 | 1 | 0 | 6 |
| De Roos et al.2005c | 1 | 0 | 1 | 0 | 1 | 1 | 1 | 1 | 0 | 6 |
| De Roos et al.2005d | 1 | 0 | 1 | 0 | 1 | 1 | 1 | 1 | 0 | 6 |
| Cocco et al.2007a | 0 | 0 | 0 | 0 | 0 | 0 | 1 | 1 | 0 | 2 |
| Cocco et al.2007b | 0 | 0 | 0 | 0 | 0 | 0 | 1 | 1 | 0 | 2 |
| Cocco et al.2007c | 0 | 0 | 0 | 0 | 0 | 0 | 1 | 1 | 0 | 2 |

* The study quality was assessed according to the Newcastle Ottawa Quality assessment scale for case-control studies. This scale awards a maximum of 9 points to each study: 4 for selection, 2 for comparability, and 3 for assessment of outcomes (for cohort study). 1 = “Yes”, 0 = “No”, “Unable to determine” or “Not available”. For case-control studies, 1, indicates cases independently validated; 2, cases are representative of population; 3, community controls; 4, controls have no history of Non-Hodgkin Lymphoma; 5A, study controls for age; 5B, study controls for additional factor(s); 6, ascertainment of exposure by blinded interview or record; 7, same method of ascertainment used for cases and controls; and 8, nonresponse rate the same for cases and controls.
